# Laparoscopic and robotic-assisted mesh pelvic closure for locally advanced and recurrent colorectal cancer

**DOI:** 10.1093/jscr/rjab524

**Published:** 2021-11-29

**Authors:** Siti Mayuha Rusli, Jeong Min Choo, Guglielmo Niccolò Piozzi, Seon Hahn Kim

**Affiliations:** Division of Colon and Rectal Surgery, Department of Surgery, Korea University Anam Hospital, Korea University College of Medicine, Seoul, South Korea; Department of Surgery, Faculty of Medicine, Universiti Teknologi MARA, Sungai Buloh Campus, Selangor, Malaysia; Division of Colon and Rectal Surgery, Department of Surgery, Korea University Anam Hospital, Korea University College of Medicine, Seoul, South Korea; Division of Colon and Rectal Surgery, Department of Surgery, Korea University Anam Hospital, Korea University College of Medicine, Seoul, South Korea; Division of Colon and Rectal Surgery, Department of Surgery, Korea University Anam Hospital, Korea University College of Medicine, Seoul, South Korea

## Abstract

Extensive resection that may be required in locally advanced and recurrent colorectal cancer result in formation of empty pelvic cavity that has the potential to cause small bowel descent into the pelvis. In patients with prior history of radiotherapy and multiple abdominal surgery, the risk of adhesion and subsequent small bowel obstruction can lead to increasing need for surgery and its resulting morbidity and mortality. We present five cases of locally advanced and recurrent colorectal cancer requiring laparoscopic and robotic-assisted pelvic closure with Gore-Tex Dual Mesh as prevention of small bowel descent into the pelvis. One out of the five cases had a history of small bowel obstruction after the surgery and wound-related infection occurred in one patient. There was no mesh-related complication or mortality. Pelvic closure using Gore-Tex Dual Mesh is feasible to prevent small bowel descent after surgery for locally advanced and recurrent colorectal cancer.

## INTRODUCTION

Patients with history of preoperative radiation have increased risk of late small bowel obstruction and abdominal pain requiring recurrent admissions [1–2]. These risks are further augmented in patients who had previous abdominal surgery [3]. In these patients, management of bowel obstruction from prolapsed bowel within the empty pelvis formed after extensive resection in locally advanced or recurrent colorectal cancer can be troublesome with increased morbidity and mortality. Various methods have been employed to eliminate the dead space and to reduce this risk. To date, there is no one superior method over another.

We present five cases where pelvic closure was performed using Gore-Tex Dual Mesh (WL Gore & Associates, Inc., Newark, DE, USA) in fully laparoscopic and robotic-assisted approach to reduce the risk of small bowel descent. All cases were performed by a single consultant colorectal surgeon between November 2019 to December 2020. After adhesiolysis and bowel resection, small bowels were placed in abdominal cavity. Gore-Tex Dual Mesh (10 × 15cm) placement at the pelvic inlet was performed laparoscopically in all cases except 1 with robotic assistance. The microporous smooth surface faced the abdominal cavity and the macroporous rough surface faced the pelvic cavity. The mesh was anchored to sacral promontory, pelvic brim and lateral peritoneum using tacker and reinforced intracorporeally with polypropylene 2/0 or endoclip with 1.0–1.5 cm gap between the sutures. The mesh was extended 2–3 cm beyond the edge of pelvic inlet. Placement of the mesh took an additional 30 minutes of operating time. Pelvic drain was inserted either transabdominally or transanally. Follow-up included history of bowel obstruction after surgery or evidence of small bowel descent within the pelvis from CT abdomen performed for evaluation of disease. Wound infection and other surgical related complications were also assessed.

## CASE SERIES

### Case 1

A 45-year-old man underwent robotic-assisted intersphincteric resection with covering ileostomy for a moderately differentiated adenocarcinoma (ypT3N0M0) low rectal cancer following long-course neoadjuvant chemoradiotherapy. Circumferential margin was 1 mm with evidence of perforation. He completed adjuvant chemoradiotherapy (FOLFOX) and underwent ileostomy reversal 6 months later. Over the next 3 years, he complained of perianal pain and discharge due to anastomotic stricture and chronic fistula with pelvic abscess. Sigmoidoscopy showed no evidence of tumour recurrence. Serial CT abdomen and MRI pelvis showed increasing presacral fluid collection. He underwent laparoscopic ultra-low Hartmann’s operation. Intraoperatively, there was severe pelvic inflammation and pus within presacral space was drained. Pelvic dissection was performed laparoscopically until the anus and dissection was completed transanally within intersphincteric space. After bowel transection, specimen was delivered transanally and hand-sewn closure of anal mucosa was performed. Laparoscopic application of the mesh was performed after copious irrigation of pelvic cavity and placement of transabdominal pelvic drain followed by formation of end colostomy. He stayed for 38 days after surgery due to complication from iatrogenic bladder injury requiring bilateral percutaneous nephrostomy. After follow-up of 17.5 months, there was a history of admission to another hospital for small bowel obstruction due to adhesion that did not require surgical intervention.

### Case 2

**Figure 1:**
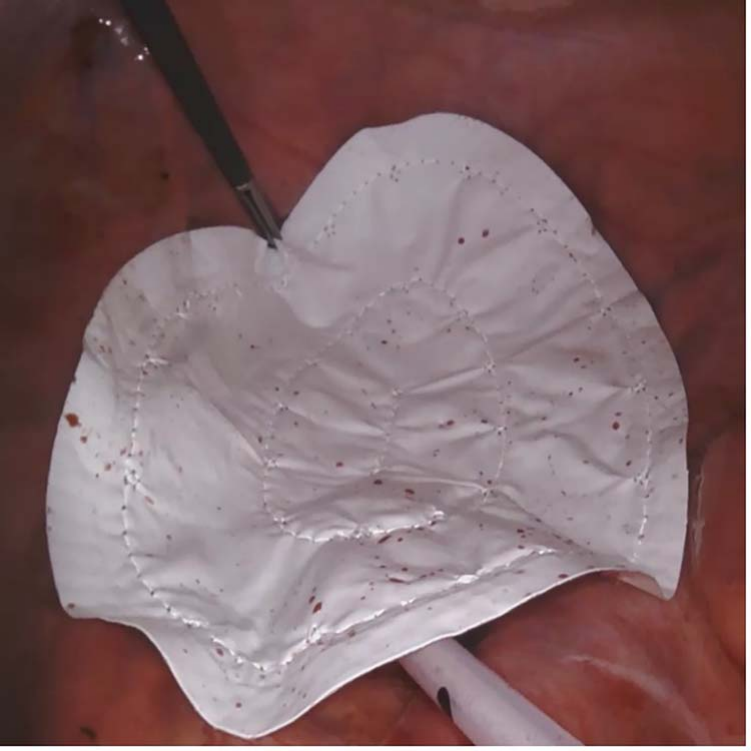
Case 2—laparoscopic picture of Gore-Tex Dual Mesh placed at the pelvic inlet with the microporous smooth side facing the abdominal cavity.

A 42-year-old man with perianal abscess and fistula due to low rectal cancer had completed long-course radiotherapy but was unable to complete FOLFIRI regimen due to elevated liver enzymes because of his underlying chronic hepatitis B. He underwent robotic-assisted abdominoperineal resection (APR) 6 weeks later. Mesh placement was performed laparoscopically after placing transabdominal pelvic drain (Fig. 1). He developed perineal wound infection on postoperative day 4 requiring wound dressings and antibiotics. He was discharged 17 days after surgery but was readmitted 28 days after initial operation for presacral abscess requiring percutaneous drainage. Subsequent CT abdomen showed resolution of presacral collection. Histopathological examination showed moderately differentiated adenocarcinoma ypT3N0M0. He completed adjuvant capecitabine and after 12.5 months, there was no evidence of unhealed perineal wound or small bowel descent.

### Case 3

**Figure 2:**
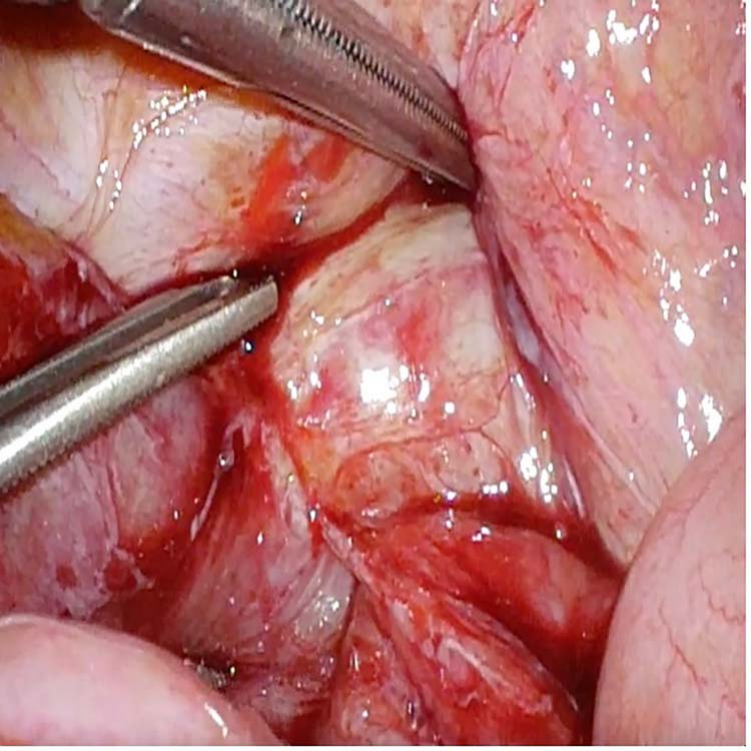
Case 3—small bowel loop adherent to anastomotic recurrence.

**Figure 3:**
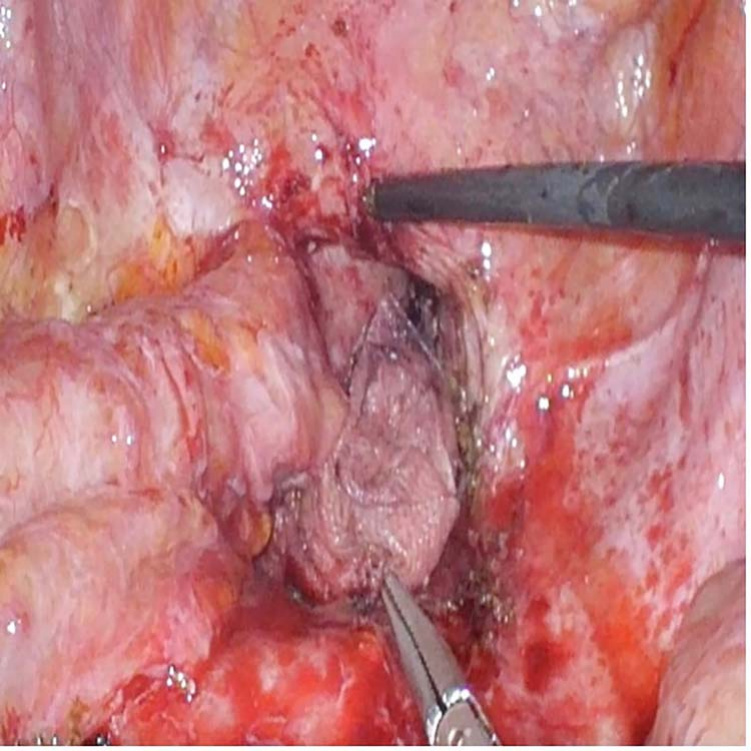
Case 3—unresectable anastomotic recurrence after resection of adherent small bowel.

A 72-year-old man underwent open low anterior resection with covering ileostomy for mid rectal cancer at another hospital after completion of long-course neoadjuvant chemoradiotherapy. Ileostomy reversal was performed 3 months after surgery. He developed intestinal obstruction secondary to anastomotic recurrence 6 months later. Sigmoidoscopy and biopsy confirmed the presence of recurrent adenocarcinoma. CT abdomen showed infiltrating soft tissue mass at right pelvic cavity with involvement of presacral area and ileal loop. He was admitted to our centre and underwent laparoscopic small bowel resection. Intraoperatively, small bowel was adherent to pelvic cavity and site of recurrence resulting in proximal small bowel dilatation (Fig. 2). Small bowel adherent to the recurrence site was resected and side-to-side small bowel anastomosis was performed. The anastomotic recurrence was unresectable (Fig. 3). Transabdominal pelvic drain was inserted followed by mesh placement. He was discharged after 8 days and after a follow-up of 12.1 months, did not develop further small bowel obstruction.

### Case 4

A 64-year-old woman with history of total abdominal hysterectomy and bilateral salphingo-oophorectomy was diagnosed with sigmoid colon cancer with vaginal invasion and lung metastases. After 4 cycles of FOLFIRI and cetuximab, she underwent robotic-assisted low anterior resection and partial vaginectomy. She completed 12 cycles of FOLFIRI and cetuximab. Over the years, she developed systemic disease progression requiring several changes of chemotherapy and targeted therapy regimen with resection of lung and liver metastases. Two years after initial diagnosis, she developed peritoneal and pelvic recurrence. She underwent radiotherapy for pelvic recurrence and was started on oral neucloside TAS-102 after her disease progressed further. She developed recurrent mechanical obstruction 1 year after radiotherapy and underwent laparoscopic small bowel resection. Intraoperatively, iatrogenic perforation of the distended small bowel occurred during laparoscopic port access and was repaired with absorbable suture. The tumour had invaded the bladder and an adherent loop of small bowel had caused proximal bowel dilatation. Small bowel transection with side-to-side small bowel anastomosis was performed followed by mesh placement. Two transabdominal drains were inserted within pelvic cavity; 1 below and 1 above the mesh. There was no postoperative complication related to the surgery. She was discharged after 15 days. After a follow-up of 6.6 months, there was no recurrence of small bowel obstruction.

### Case 5

A 58-year-old man with obstructed upper rectal cancer underwent laparoscopic low anterior resection with covering ileostomy at another hospital. Ileostomy reversal was subsequently performed. He only completed seven cycles of FOLFOX due to oxaliplatin-induced persistent peripheral neuropathy. He developed anastomotic stricture due to pelvic recurrence and lung metastases as confirmed by sigmoidoscopy and CT thorax, abdomen and pelvis 9 months after his treatment necessitating transverse loop colostomy. He underwent chemoradiotherapy with capecitabine that was later changed to FOLFIRI and bevacizumab. He subsequently underwent robotic-assisted Hartmann’s procedure due to persistent pelvic pain and abscess. Intraoperatively, there was pelvic abscess due to disrupted neorectum from the pelvic recurrence. The hostile pelvic environment did not allow further dissection and proximal bowel up to the disrupted neorectum was then transected followed by formation of end colostomy. After copious irrigation of the pelvic cavity, mesh was applied using robotic assistance. Transanal drain was inserted into the opened distal neorectum lumen. There was no wound complication, and he was discharged after 31 days as he had to undergo several ganglion nerves blocks for pain management. After a follow-up of 5.2 months, there was no occurrence of small bowel obstruction.

## DISCUSSION

Radical resection for locally advanced and recurrent rectal cancer results in extensive tissue loss leading to formation of cavity within the pelvis that can lead to small bowel descent. Pelvic closure was initially performed to prevent small bowel descending into the pelvis that may be within the radiation field and to optimise the dosage and delivery of radiotherapy after surgery. As neoadjuvant chemoradiotherapy became the standard of care for locally advanced rectal cancer and surgical advancement like extralevator abdominoperineal excision was introduced, pelvic closure was performed together with perineal reconstruction to reduce the associated pelviperineal morbidity.

Various types of mesh were used as a sling to prevent small bowel prolapse. Devereux et al. had described the use of absorbable polyglycolic acid mesh in 60 patients with rectal and pelvic cancers and found no patient with radiation enteritis or mesh-related complications [4]. A study looking at complications following polyglycolic acid and polyglactin 910 mesh placement after rectal cancer surgeries found that complications that may be related to mesh placement in immediate postoperative period were comparable to those performed prior to mesh placement with late bowel obstruction occurring in 1 out of 20 patients with mesh [5]. Waddell et al. noted that patients with rectal cancer who underwent APR and had either polyglycolic acid or polyglactin 910 mesh sling anchored were able to receive full dose of radiation with no evidence of late gastrointestinal effects [6]. Absorbable mesh made of polyglycolic acid and trymethylene copolymers have been used in patients who had undergone APR and apart from one patient who developed chronic pelvic pain, no abdominal or perineal complications were seen after a mean follow up of 9 months [7]. Although complications related to absorbable mesh are acceptable absorbable mesh may lose the tensile strength needed to prevent small bowel descent into the pelvis. Polyglactin 910 mesh loses 50% of its mechanical stability after 3 weeks and the newly formed connective tissue loses its mechanical strength completely after 6 weeks [8].

Considering that survival rate for advanced colorectal cancer is improving over time, usage of non-absorbable mesh may be useful in preventing small bowel descent into the pelvis. Non-absorbable mesh has less retraction, and the mechanical strength can be up to 10 times that of absorbable mesh [9]. The issues that are usually raised with the usage of non-absorbable mesh are related to presence of foreign body and its associated complications including infection, obstruction and fistula. They are widely used in repair of abdominal wall and inguinal hernia but are less commonly used in intraabdominal or pelvic surgeries. Three of the most used non-absorbable mesh are made of polyester, polypropylene and expanded polytetrafluoroethylene (ePTFE). Polyester mesh is associated with high incidence of complications and is not commonly used now [10]. Reports of erosion and fistula with usage of polypropylene mesh in pelvic surgeries could be related to higher degree of adhesion found on the macroporous polypropylene mesh [11–12]. ePTFE material is used when there is risk of adhesion to surrounding viscera. It was shown to be safe in preventing adhesion after pelvic surgery [13]. Gore-Tex Dual Mesh has two different functional surfaces. The textured surface allows host tissue incorporation, while the smooth surface reduces tissue reaction. It has been used in repair of ventral and parastomal hernia with acceptable rate of mesh-related complications [14–15]. When used as pelvic floor support for vaginal vault prolapse, Clavero et al. experienced mesh-related infection in 1 out of 16 cases [16]. A modified usage of the mesh applying the smooth surfaces of two pieces of Gore-Tex Dual Mesh to isolate both the abdominal and pelvic cavities in a patient with recurrent enterovesicocervical fistula showed no recurrence after a follow up period of 18 months [17]. To the best of our knowledge, only one study investigated the usage of ePTFE mesh for pelvic closure after rectal cancer surgery. Cui et al. compared the short-term and long-term outcome of patients with rectal cancer who had undergone APR with and without Gore-Tex Dual Mesh pelvic floor reconstruction [18]. Those with mesh reconstruction had better short-term outcomes and no incidence of bowel obstruction. Our cases differ in that all mesh placements were performed in fully laparoscopic and robotic-assisted manner.

Some consideration when placing the mesh include the care taken not to injure the ureter, major pelvic vessels and neurovascular bundle. When placing the mesh within infected pelvic cavity, copious irrigation and elimination of pelvic abscess and placement of pelvic drain to reduce reaccumulation of fluid and pus are essential. Although we do not advocate routine mesh closure of the pelvis after primary APR, patients who are at higher risk of small bowel obstruction with subsequent morbidity associated with it may benefit from pelvic closure using Gore-Tex Dual Mesh. Perioperative complications were related to disease process rather than mesh placement itself and none of the patients required further surgery.

## References

[1] Chessin DB , Enker W, Cohen AM, Paty PB, Weiser MR, Saltz L, et al. Complications after preoperative combined modality therapy and radical resection of locally advanced rectal cancer: a 14-year experience from a specialty service. J Am Coll Surg 2005;200:876–82. 15922198 10.1016/j.jamcollsurg.2005.02.027

[2] Birgisson H , Påhlman L, Gunnarsson U, Glimelius B. Late gastrointestinal disorders after rectal cancer surgery with and without preoperative radiation therapy. Br J Surg 2008;95:206–13. 17849380 10.1002/bjs.5918

[3] Ha GW , Lee MR, Kim JH. Adhesive small bowel obstruction after laparoscopic and open colorectal surgery: a systematic review and meta-analysis. Am J Surg 2016;212:527–36. 27427294 10.1016/j.amjsurg.2016.02.019

[4] Devereux DF , Chandler JJ, Eisenstat T, Zinkin L. Efficacy of an absorbable mesh in keeping the small bowel out of the human pelvis following surgery. Dis Colon Rectum 1988;31:17–21. 2835216 10.1007/BF02552563

[5] Beitler A , Rodriguez-Bigas MA, Weber TK, Lee RJ, Cuenca R, Petrelli NJ. Complications of absorbable pelvic mesh slings following surgery for rectal carcinoma. Dis Colon Rectum 1997;40:1336–41. 9369109 10.1007/BF02050819

[6] Waddell BE , Lee RJ, Rodriguez-Bigas MA, Weber TK, Petrelli NJ. Absorbable mesh sling prevents radiation-induced bowel injury during “sandwich” chemoradiation for rectal cancer. Arch Surg 2000;135:1212–7. 11030884 10.1001/archsurg.135.10.1212

[7] Moreno-Sanz C , Manzanera-Díaz M, Cortina-Oliva F, de Pedro-Conal J, Clerveus M, Picazo-Yeste J. Pelvic reconstruction after abdominoperineal resection: a pilot study using an absorbable synthetic prosthesis. Tech Coloproctol 2011;15:455–9. 21960412 10.1007/s10151-011-0763-8

[8] Klinge U , Schumpelick V, Klosterhalfen B. Functional assessment and tissue response of short- and long-term absorbable surgical meshes. Biomaterials 2001;22:1415–24. 11336316 10.1016/s0142-9612(00)00299-4

[9] Boukerrou M , Boulanger L, Rubod C, Lambaudie E, Dubois P, Cosson M. Study of the biomechanical properties of synthetic mesh implanted in vivo. Eur J Obstet Gynecol Reprod Biol 2007;134:262–7. 17459566 10.1016/j.ejogrb.2007.02.023

[10] Leber GE , Garb JL, Alexander AI, Reed WP. Long-term complications associated with prosthetic repair of incisional hernias. Arch Surg 1998;133:378–82. 9565117 10.1001/archsurg.133.4.378

[11] Kobashi K , Govier F. Management of vaginal erosion of polypropylene mesh slings. J Urol 2003;169:2242–3. 12771759 10.1097/01.ju.0000060119.43064.f6

[12] Kiudelis M , Jonciauskiene J, Deduchovas O, et al. Effects of different kinds of meshes on postoperative adhesion formation in the New Zealand white rabbit. Hernia 2007;11:19–23. 16977345 10.1007/s10029-006-0139-6

[13] Hurst BS , et al. Permanent implantation of expanded polytetrafluoroethylene is safe for pelvic surgery. Hum Reprod 1999;14:925–7. 10221220 10.1093/humrep/14.4.925

[14] Topart P , Ferrand L, Vandenbroucke F, Lozac’h P. Laparoscopic ventral hernia repair with the Goretex Dualmesh: long-term results and review of the literature. Hernia 2005;9:348–52. 16012779 10.1007/s10029-005-0013-y

[15] Hansson BME , de Hingh IHJ, Bleichrodt RP. Laparoscopic parastomal hernia repair is feasible and safe: early results of a prospective clinical study including 55 consecutive patients. Surg Endosc 2007;21:989–93. 17353985 10.1007/s00464-007-9244-6

[16] Clavero PA , Guerrero JA, Salamanca A. Gore-Tex mesh pelvic occlusion and secondary colpopexy: a new surgical technique for posthysterectomy vaginal vault prolapse. Eur J Obstet Gynecol Reprod Biol 2006;126:113–5. 16684626 10.1016/j.ejogrb.2005.07.032

[17] Leandros E , Antonakis PT, Gomatos I, Tsigris C, Konstadoulakis MM. Pelvic isolation with two Gore-tex dual mesh pieces for a recurrent complicated enterovesicocervical fistula in a patient irradiated for cervical cancer. Am Surg 2009;75:1146–8. 19927526

[18] Cui J , Ma JP, Xiang J, et al. Prospective study of reconstructing pelvic floor with GORE-TEX dual mesh in abdominoperineal resection. Chin Med J 2009;122:2138–41. 19781299

